# Hodgkin’s lymphoma characteristics in HIV-infected and uninfected patients at an urban hospital in the late combined antiretroviral era

**DOI:** 10.1186/1750-9378-7-S1-P26

**Published:** 2012-04-19

**Authors:** Clifford Gunthel, Marylin Adamski, Marina Mosunjac, Minh Ly Nguyen

**Affiliations:** 1 Division of Infectious Diseases, Emory University School of Medicine, Atlanta, GA, USA; 2 Infectious Disease Program, Grady Health System, Atlanta, GA, USA; 3 Department of Pathology, Emory University School of Medicine, Atlanta, GA, USA

## Background

As combined antiretroviral therapy has allowed patients infected with HIV to survive longer due to improved immunity, increasing incidences of non-AIDS associated malignancies as well as chronic comorbidities are reported. One of the more commonly reported non-AIDS associated cancers is Hodgkin’s lymphoma (HL) [1]. We report our experience of HL among HIV-infected and uninfected patients.

## Methods

Grady Health System (GHS) provides care to the majority of the urban indigent population of Atlanta. Patients who were diagnosed with HL between January 2000 and June 2011were identified from the GHS pathology records and the GHS cancer registry. Clinic charts and medical records were reviewed. Patients’ demographics, CD4 counts, HIV viral load, HIV and HL treatment and outcomes were recorded.

## Results

During the study period, 95 patients were diagnosed with HL (26% HIV-, 30% HIV+ and 43% HIV status unknown). The characteristics are displayed in Table 1.

**Table 1:** Characteristics of HL in HIV- and HIV + patients

	HIV-	HIV+
N	25	29
M:F	16:9	22:7
Race (black:other)	20:5	25:4
Median age (range)	33(19-52)	40(22-54)
Stage (I-II versus III-IV)	5:9	2:19
B symptoms	4	5
Diagnosis made solely by bone marrow biopsy	0	3
Morphology NS/LR versus MC/LD	12:5	9:2
One year survival	76%	45%

Among the HIV+ patients, at time of HL diagnosis, the median Cd4 at time of HL diagnosis was 95(8-865)cells/mm^3^, and 3 (10%) are on cART .The median time from HIV diagnosis to HL diagnosis is 2 years (0-20).

## Conclusions

In the current cART era, in our institution, HL in HIV+ patients is more likely to present with advanced disease (65% with stage III/IV). Interestingly, in 3 HIV+ patients, HL was diagnosed solely by bone marrow biopsy. Despite the availability of cART, patients are not accessing care. This may account for the poor one-year survival among HIV+ patients with HL.
